# Non-classical presentation of vitamin D deficiency: a case report

**DOI:** 10.1186/s13256-020-02454-1

**Published:** 2020-08-10

**Authors:** Mohanad Kamaleldin Mahmoud Ibrahim, Mustafa Khidir Mustafa Elnimeiri

**Affiliations:** 1 Community Medicine and Epidemiology, Faculty of Medicine, Ibn Sina University, Khartoum, Sudan; 2 Preventive Medicine and Epidemiology, Alneelain University, Khartoum, Sudan

**Keywords:** Vitamin D deficiency, Allergy, Nasal polyps, Backache, Chronic fatigability

## Abstract

**Background:**

Vitamin D is a fat-soluble vitamin; vitamin D is essential to sustain health and it protects against osteoporosis. It is crucial to the human body’s physiology in terms of muscular movement and neurological signal transmission, and to the immune system in defense against invading pathogens.

**Case presentation:**

This was a case of a 26-year-old Sudanese woman who presented with a 2-year history of anosmia, recurrent nasal polyps, back pain, and chronic fatigue. She was diagnosed as having a case of vitamin D deficiency and responded well to treatment.

**Conclusion:**

There is an association between vitamin D deficiency and recurrent allergic nasal conditions.

## Background

Vitamin D is a fat-soluble vitamin; it is naturally present in some foods and as dietary supplements. It is also produced endogenously through exposure to ultraviolet rays from sunlight. Vitamin D obtained from sun exposure, food, and supplements is biologically inert and must undergo two hydroxylations in the body for activation. The first occurs in the liver and produces 25-hydroxyvitamin D (25(OH)D), also known as calcidiol. The second occurs in the kidney and forms the physiologically active 1,25-dihydroxy vitamin D (1,25(OH)_2_D), also known as calcitriol [1].

Vitamin D is found in cells throughout the body; vitamin D is essential to sustain health and it protects against osteoporosis. It is crucial to the human body’s physiology in terms of muscular movement and neurological signal transmission, and to the immune system in defense against invading pathogens [2].

Although there are different methods and criteria for defining vitamin D levels, the criteria Holick proposed have been widely accepted. In this proposal, vitamin D deficiency is defined as blood level of less than 20 ng/ml; insufficiency of vitamin D is defined as blood levels ranging between 20 and 29.9 ng/ml and sufficiency if greater than or equal to 30 ng/ml [3]. About one billion people globally have vitamin D deficiency and 50% of the population has vitamin D insufficiency. The majority of affected people with vitamin D deficiency are the elderly, obese patients, nursing home residents, and hospitalized patients. Vitamin D deficiency arises from multiple causes including inadequate dietary intake and inadequate exposure to sunlight. Certain malabsorption syndromes such as celiac disease, short bowel syndrome, gastric bypass, some medications and cystic fibrosis may also lead to vitamin D deficiency [4].

Vitamin D deficiency is now more prevalent than ever and should be screened in high-risk populations. Many conflicting studies now show an association between vitamin D deficiency and cancer, cardiovascular disease, diabetes, autoimmune diseases, and neuropsychiatric disorders [5, 6].

## Case presentation

This was a case of a 26-year-old Sudanese woman, married, who has a 3-year-old boy. This woman presented to our ear, nose, and throat (ENT) department complaining of anosmia for the past 2 years. She had a history of two functional endoscopic sinus surgeries (FESSs) for nasal polyps: the first one was 6 years ago and the second one was 3 years prior to presentation. She complained of being highly sensitive to different irritants including dust, weather change, perfumes, and pets.She also stated that she attended more than three different physicians due to generalized fatigue and getting tired easily after simple daily activity in addition to sleeping for more than 10 hours a day.She attended an orthopedic clinic for unspecified lower back pain that was not related to any type of trauma or physical activity; a lumbosacral magnetic resonance imaging (MRI) was done and revealed no abnormal findings.She mentioned that she is known to be anxious most of the time and aggressive toward simple reactions from her family members. She had no psychiatric history and was not using any medications.

She was not known to be diabetic or hypertensive or to have any chronic illnesses; she was not on any regular medication. She is a housewife of high socioeconomic status; she is well educated, graduated from dental school with a bachelor’s degree, but currently not employed. She has never consumed tobacco or alcohol; she practiced regular cardio exercises.On examination, she looked healthy, well, not pale or jaundiced. Her pulse rate was 74/minute and her blood pressure was 118/70. Her body mass index (BMI) was 26.8. All systems examinations were normal except for bilateral nasal polyps. Complete blood count (CBC), renal function test (REF), electrolyte, liver function test (LFT), thyroid function test (TFT), urine analysis (general urine test), antinuclear antibody (ANA), and rheumatoid factor (RF) were all normal. An imaging profile included lumbo-sacral MRI, a computed tomography (CT) scan of her sinuses, and electrocardiogram (ECG), which were normal except for bilateral nasal polyps and severe sinusitis that looked allergic to fungi in nature.She underwent FESS surgery to remove the polyps and clean out her sinuses; up to 6 weeks after surgery she used nasal steroids (mometasone furoate 0.005%) two times a day, but her symptoms regarding anosmia were not improved. MRI of her brain and a CT scan of her sinuses were done and both revealed normal features. A vitamin D deficiency was suggested and the laboratory results revealed a low vitamin D level of 7 ng/ml. Treatment with vitamin D supplement was prescribed at 50,000 international units (IU) weekly for 8 weeks and then 1000 IU maintenance dose daily, she was advised to take food rich in vitamin D and get exposed to sunlight for 20 minutes three times a week after the loading dose of supplement. She was at regular follow-up for 6 months; at rates of weekly for the first month, every 2 weeks for the second month, and monthly for the rest of the follow-up period. At each visit, she was assessed with clinical history and examination. It was noticed that the symptoms of tiredness, sleeping, anosmia, and back pain were dramatically improving during that period. At the 6 months follow-up, her blood level of vitamin D was normal, she described her condition as free from all symptoms, and she returned back to normal physical activity.

## Discussion and conclusions

This was a non-classical case of vitamin D deficiency of a 26-year-old woman who presented with chronic anosmia and recurrent nasal polyps. She was diagnosed as having a case of vitamin D deficiency and responded well to vitamin D replacement therapy. This case correlated an association between decreased levels of vitamin D and recurrent nasal polyps that led in time to chronic anosmia as a result of chronic high sensitivity reactions triggered by our patient’s autoimmune system. The literature links chronic rhinosinusitis with nasal polyps (CRSwNP) with asthma and allergic rhinitis, but the cellular and molecular mechanisms that contribute to the clinical symptoms are not fully understood. Sinonasal epithelial cell barrier defects, increased exposure to pathogenic and colonized bacteria, and dysregulation of the host immune system are all thought to play prominent roles in disease pathogenesis [7].

Despite all the previous surgical and medical interventions over the past 6 years, our patient’s condition did not improve and she still complained of anosmia. A study revealed that this patient was experiencing excessive allergic reactions that led to recurrent nasal polyps. It is well known that classical clinical effects of vitamin D deficiency are bones and musculoskeletal-related disorders, several lines of evidence demonstrate the effects of vitamin D on pro-inflammatory cytokines, regulatory T cells, and immune responses, with a conflicting interpretation of the effects of vitamin D on allergic diseases [8].

The working diagnosis was suggested in relation to some musculoskeletal symptoms and chronic fatigue especially when the imaging profile for her lower back and all routine investigations were normal. It has been suggested that clinicians should routinely test for hypovitaminosis D in patients with musculoskeletal symptoms, such as bone pain, myalgias, and generalized weakness which might be misdiagnosed as fibromyalgia and chronic fatigue [9]. The most common causes of anosmia were assessed as well and they were negative, these included sinonasal diseases, post infectious disorder, and post-traumatic disorder, and congenital defects and disorders caused by neurodegenerative disease [10].

Thus blood level for vitamin D was requested and the results were of low D level.

In the past history of the previous nasal polyps surgeries, our patient noted that there was no anosmia and her main complaints were classic complaints of sinusitis, including sneezing, nasal blockage and headache. Soon after surgery her symptoms improved except for the allergy-related symptoms, despite usage of inhaled steroids spray. She stated that, at the last time, the presentation was different since it was only anosmia, indicating that there was significant inflammation that affected the smell receptors around the olfactory epithelium. After the last nasal polyps and sinuses drainage surgery, the symptoms related to allergic reactions, including chronic sneezing, did not improve for up to 6 weeks and she was still suffering from hyposmia, although that was a fair postoperative period for recovery.

The symptoms of anosmia and sneezing, and other systematic symptoms, gradually started to improve after vitamin D supplements, indicating that the main reason behind her symptoms was vitamin D deficiency. She was followed up for up to 6 months after establishment of vitamin D supplements and at the last follow-up she had a normal sense of smell, and she was free from back pain, fatigue, and allergy-related symptoms.

This was a non-classical presentation as our patient was young and she did not have alkaline phosphatase, calcium, and phosphorus abnormalities [11] that are expected in cases of vitamin D deficiency.

This case revealed an association between decreased levels of vitamin D and recurrent nasal polyps that led to anosmia as a result of hypersensitive reactions produced by the body’s systems.

Although vitamin D deficiency is prevalent, measurement of serum 25(OH)D level is expensive, and universal screening is not supported. However, vitamin D testing may benefit those at risk for severe deficiency.

It is highly recommended to consider vitamin D deficiency among all patients with unspecified symptoms or in cases of non-diagnosed disorder regardless of the presenting complaint.

In conclusion, there is an association between vitamin D deficiency and recurrent allergic nasal conditions.

## Data Availability

The datasets used and/or analyzed during the current study are available from the corresponding author on reasonable request.

## Abbreviations

CRSwNP: Chronic rhinosinusitis with nasal polyps
CT: Computed tomography
ECG: Electrocardiogram
ENT: Ear, nose, and throat
FESS: Functional endoscopic sinus surgery
BMI: Body mass index
CBC: Complete blood count
RFT: Renal function test
LFT: Liver function test
TFT: Thyroid function test
ANA: Antinuclear antibody
RF: Rheumatoid factor
IU: International unit
